# Inhibiting KLRB1 expression is associated with impairing cancer immunity and leading to cancer progression and poor prognosis in breast invasive carcinoma patients

**DOI:** 10.18632/aging.205239

**Published:** 2023-11-20

**Authors:** Jin-Rong He, Dan Li, Qun-Xian Zhang, Tao Liu, Yu Ding, Chuang-Yan Wu, Shan-Shan Chen, Jiu-Ling Chen

**Affiliations:** 1 Key Laboratory for Molecular Diagnosis of Hubei Province, The Central Hospital of Wuhan, Tongji Medical College, Huazhong University of Science and Technology, Wuhan 430014, Hubei, China; 2 Department of Oncology, Taihe Hospital, Hubei University of Medicine, Shiyan 442012, Hubei, China; 3 Department of Cardiothoracic Surgery, Taihe Hospital, Hubei University of Medicine, Shiyan 442012, Hubei, China; 4 Department of Thoracic Surgery, Union Hospital, Tongji Medical College, Huazhong University of Science and Technology, Wuhan 430022, Hubei, China

**Keywords:** KLRB1, BRCA, immune microenvironment, biomarker, prognosis

## Abstract

Background: The association between Killer cell lectin like receptor B1 (KLRB1) and cancer has been reported, but the roles of KLRB1 in breast invasive carcinoma (BRCA) has not been fully revealed.

Methods: Our study utilized the Cancer Genome Atlas (TCGA), Kaplan-Meier (K-M) Plotter, and TIMER databases to investigate the expression and clinical relevance of KLRB1 in BRCA and to explore its roles and mechanism in BRCA progression using gene set enrichment analysis, CCK-8, migration, apoptosis, and western blotting. We examined the relationship between KLRB1 expression and the BRCA immune microenvironment, using data from TCGA, and Gene Expression Profiling Interactive Analysis (GEPIA) databases and validated these findings in K-M Plotter databases.

Results: A significant decrease of KLRB1 expression was observed in BRCA patients. BRCA patients with low KLRB1 levels were associated with older age, advanced disease stage, HER2-positivity, poor prognosis, and a decreased survival probability compared to the high-expression group. Increased KLRB1 expression levels were correlated with inhibition of breast cancer cell proliferation, migration, and invasion, as well as promotion of cell apoptosis, possible through regulation of the NF-κB, PI3K/AKT, and TNF signaling pathways. Moreover, the study also indicated that decreased KLRB1 expression correlated with tumor purity, immune score, and immune cell infiltration (B cells, CD8^+^ T cells, CD4^+^ T cells, neutrophils, dendritic cells, among others), cell markers, and immunotherapy.

Conclusion: Decreased KLRB1 expression in BRCA is associated with poor prognosis and immune microenvironment. This study also highlights KLRB1 as a potential molecular marker for poor prognosis in BRCA patients, and therefore, it may provide clinical implications for the management of patients with BRCA.

## INTRODUCTION

Breast cancer (BC) is a prevalent malignancy among women worldwide, posing a substantial risk to their lives [1–4]. Breast invasive carcinoma (BRCA) is a subtype of BC commonly observed. Current research confirms that BRCA is most commonly the result of a combination of genetic and non-genetic factors [4–6]. Furthermore, the incidence of BRCA increases with age [7], and patients diagnosed with BRCA have a generally poor prognosis. Therefore, early diagnosis rates for BRCA are of tremendous significance in achieving early treatment and better prognosis for patients with BRCA.

Studies have reported a significantly higher number of differentially expressed genes during BRCA progression [8–10]. For instance, DEAD-box helicase 27 (DDX27) expression levels have been noted to be significantly elevated in BC tissues, with increased expression being associated with various adverse prognostic factors including tumor size, positive lymph nodes, high grade, Ki-67, pathological stage, and poor prognosis in BC patients. DDX27 overexpression has also been shown to promote stem cell-like activity, proliferation, and migration in BC cells, as well as enhance the expression of stem cell biomarkers [9]. In recent years, killer cell lectin-like receptor B1 (KLRB1) encoded by the CD161 gene has been identified, with expression in both NK cells and T cell subset, and has been found to play a significant role in cancer progression [11–13]. Studies have shown that KLRB1 gene inactivation enhances T-cell-mediated killing of glioma cells *in vitro* and the anti-tumor function of KLRB1 *in vivo* [13], whereas decreased KLRB1 expression has been associated with poor prognosis in BC patients [11]. Xu and Huang et al. found that KLRB1 is in the down-regulation trend in BC and a negative correlation between overexpression of KLRB1 and poor prognosis (such as overall survival (OS) and recurrence free survival (RFS)) of BC patients through survival analysis [14, 15]. However, not much is known about the relationship between KLRB1 expression and BC subtypes (such as the BRCA) progression. Thus, this study aimed to identify KLRB1 expression levels and their clinical significance in BRCA patients through data extracted of FPKM type from the The Cancer Genome Atlas (TCGA) database. Furthermore, we aimed to investigate the roles of KLRB1 in BRCA progression and immune microenvironment, identify its involved signaling pathways through gene set enrichment analysis (GSEA) and cellular studies (including Cell Counting Kit-8 (CCK-8), migration assay, apoptosis assay, along with other methods), and evaluate whether KLRB1 could potentially serve as a therapeutic target for BRCA patients through correlation analysis of its expression with immune microenvironment indicators of BRCA and its subtypes.

## RESULTS

### BRCA patients with significantly reduced KLRB1 expression levels exhibit poor prognosis and diagnostic values

**Figure 1:**
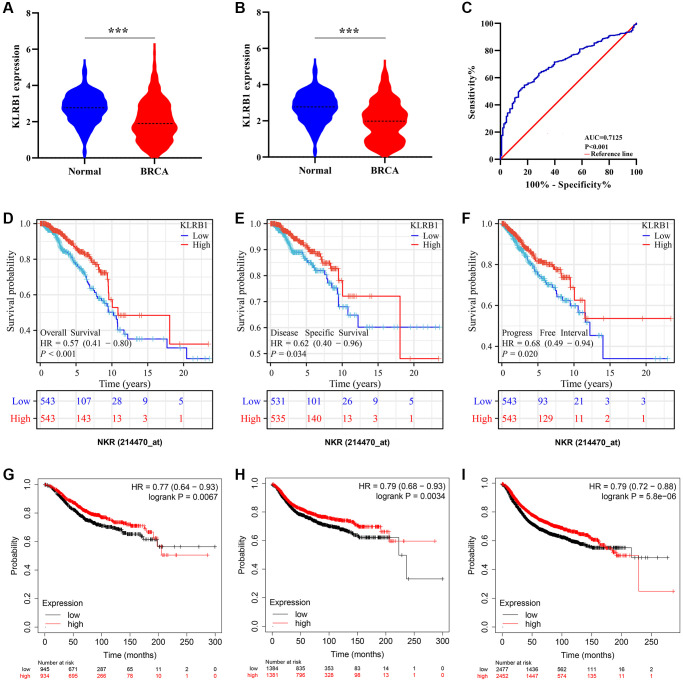
**Increased KLRB1 levels were associated with diagnosis and better prognosis in BRCA using TCGA and K-M plotter databases.** (**A**) KLRB1 expression in unpaired tissues; (**B**) KLRB1 expression in paired tissues; (**C**) The diagnostic effect of KLRB1 using ROC analysis; (**D**–**F**) The better prognosis related the KLRB1 overexpression in TCGA database by using K-M analysis; (**G**–**I**) Increased KLRB1 expression was associated with better OS, DMFS, and RFS of BRCA patients in K-M plotter database. Abbreviations: OS: overall survival; DMFS: distant metastasis-free survival; RFS: recurrence-free survival; ROC: Receiver operating characteristic; K-M: Kaplan-Meier.

KLRB1 levels were found to be significantly reduced in non-paired BRCA tissues when compared to normal breast tissues (Figure 1A). Moreover, KLRB1 expression remained at low levels in the cancer tissues of 113 paired BRCA patients (Figure 1B). Receiver operating characteristic (ROC) analysis further indicated that the area under the curve of KLRB1 was 0.7125 (Figure 1C). Furthermore, the results of Kaplan-Meier (K-M) survival analysis showed that low KLRB1 expression was significantly associated with poor prognosis, as observed in disease specific survival (DSS), OS, and progression-free interval (PFI) in BRCA patients (Figure 1D–1F). In addition, decreased KLRB1 levels were also associated with poor indicators of OS, distant metastasis-free survival (DMFS), and RFS in BRCA patients, as noted in the K-M plotter database (Figure 1G–1I).

### Reduced KLRB1 levels were linked to T stage, pathological stage, age, human epidermal growth factor receptor 2 (HER2) status, and survival status in BRCA

**Figure 2:**
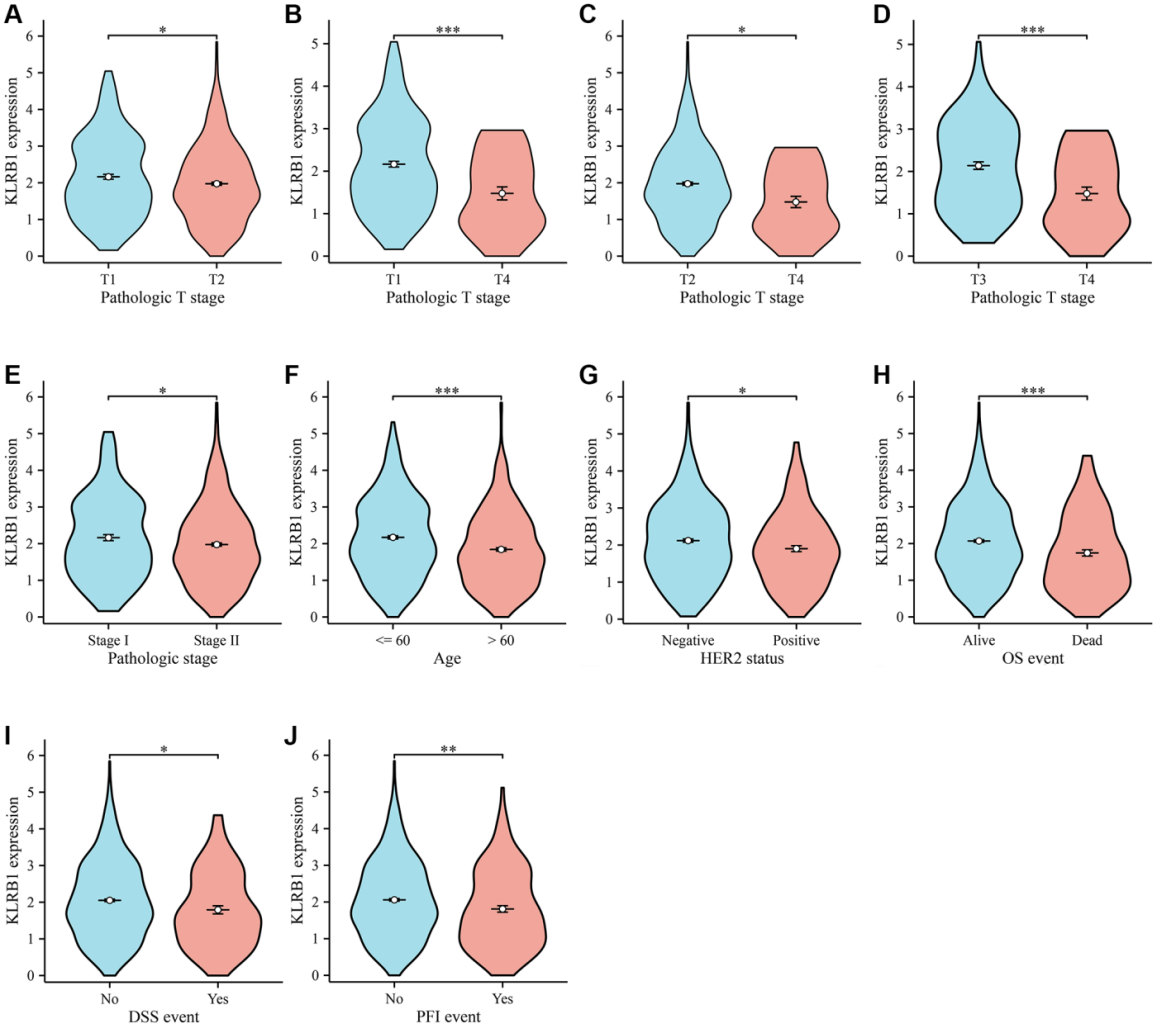
**KLRB1 in relation to clinical features in BC.** (**A**–**D**) T stage; (**E**) Pathological stage; (**F**) Age; (**G**) Her2 status; (**H**–**J**) The better prognosis related the KLRB1 overexpression.

KLRB1 expression levels were significantly decreased in tissues of BRCA patients at T2 and T4 stages compared to those at the T1 stage (Figure 2A, 2B). In addition, KLRB1 levels were prominently lower in tissues from BRCA patients with T4 stage than in patients with T2 and T3 stages (Figure 2C, 2D). Notably, KLRB1 expression levels were notably decreased in tissues of BRCA patients with pathological stage II compared to stage I, respectively (Figure 2E). KLRB1 expression level was also found to be significantly lower in tissues from patients with BRCA who were older than 60 years of age than in those 60 years of age or younger (Figure 2F). Furthermore, KLRB1 levels were significantly lower in tissues of HER2-positive BRCA patients than those with HER2-negative BRCA (Figure 2G). Additionally, KLRB1 levels were significantly reduced in tissues from patients with BRCA who had died compared to those who were alive (Figure 2H–2J).

### Decreased expression of KLRB1 was found to be strongly correlated with poor prognosis-related features in BRCA patients

In the K-M Plotter database, a survival analysis revealed that decreased KLRB1 levels were correlated with a decrease in OS, DMFS, and RFS in BRCA patients with various estrogen receptor (ER) and HER2 statuses, intrinsic and PAM50 subtypes, and lymph node statuses (Table 1). The KLRB1 levels were shown to be particularly associated with poor outcomes in BRCA patients with ER-negative or progesterone receptor (PR)-negative status, HER2-positive status, basal or luminal B intrinsic subtypes, and basal, luminal, and HER2-positive PAM50 subtypes (Table 1).

**Table 1:** The KLRB1 expression was related to the clinical features related to the prognosis of BRCA patients.

**Subtype**	**OS**			**DMFS**			**RFS**		
	***N***	**HR**	***P***	***N***	**HR**	***P***	***N***	**HR**	***P***
ER status (IHC)									
Positive	754	0.58	0.00078	1141	0.75	0.045	2633	0.75	0.00032
Negative	520	0.72	0.054	747	0.68	0.0038	1190	0.74	0.0004
ER status (Array)									
Positive	1309	0.71	0.0063	2016	0.82	0.043	3768	0.75	4.4e-06
Negative	570	0.63	0.0045	749	0.58	5.1e-05	1161	0.6	7.7e-07
PR status									
Positive	156	0.52	0.085	529	0.73	0.19	926	0.84	0.25
Negative	291	0.64	0.09	637	0.75	0.047	925	0.77	0.043
Her2 status									
Positive	420	0.46	4.6e-05	451	0.47	1.4e-05	882	0.61	1e-05
Negative	1459	0.66	0.00051	2314	0.79	0.012	4047	0.76	9.8e-06
Intrinsic subtype									
Basal	404	0.62	0.017	571	0.68	0.016	846	0.66	0.00029
Luminal A	794	0.55	0.00016	1260	0.77	0.051	2277	0.74	3e-04
Luminal B	515	0.49	0.0026	756	0.69	0.0092	1491	0.69	4.4e-05
Her2^+^	166	0.41	0.0026	178	0.32	2e-06	315	0.47	0.00012
PAM50 subtype									
Basal	431	0.47	0.00014	630	0.65	0.0058	953	0.62	4.3e-05
Luminal A	596	0.79	0.27	998	0.8	0.18	1809	1.24	0.043
Luminal B	439	0.66	0.021	673	0.64	0.0048	1353	0.71	0.00014
Her2^+^	362	0.44	0.00077	401	0.49	0.00014	695	0.57	7.2e-06
Normal	51	0.59	0.29	63	1.64	0.31	119	0.62	0.24
Lymph node status									
Yes	452	0.82	0.27	889	1.31	0.06	1656	0.86	0.088
No	726	0.75	0.11	1309	0.66	9e-04	2368	0.67	4.8e-06

### Decreased KLRB1 expression was identified as a significant risk factor for poor prognosis in BRCA patients

**Figure 3:**
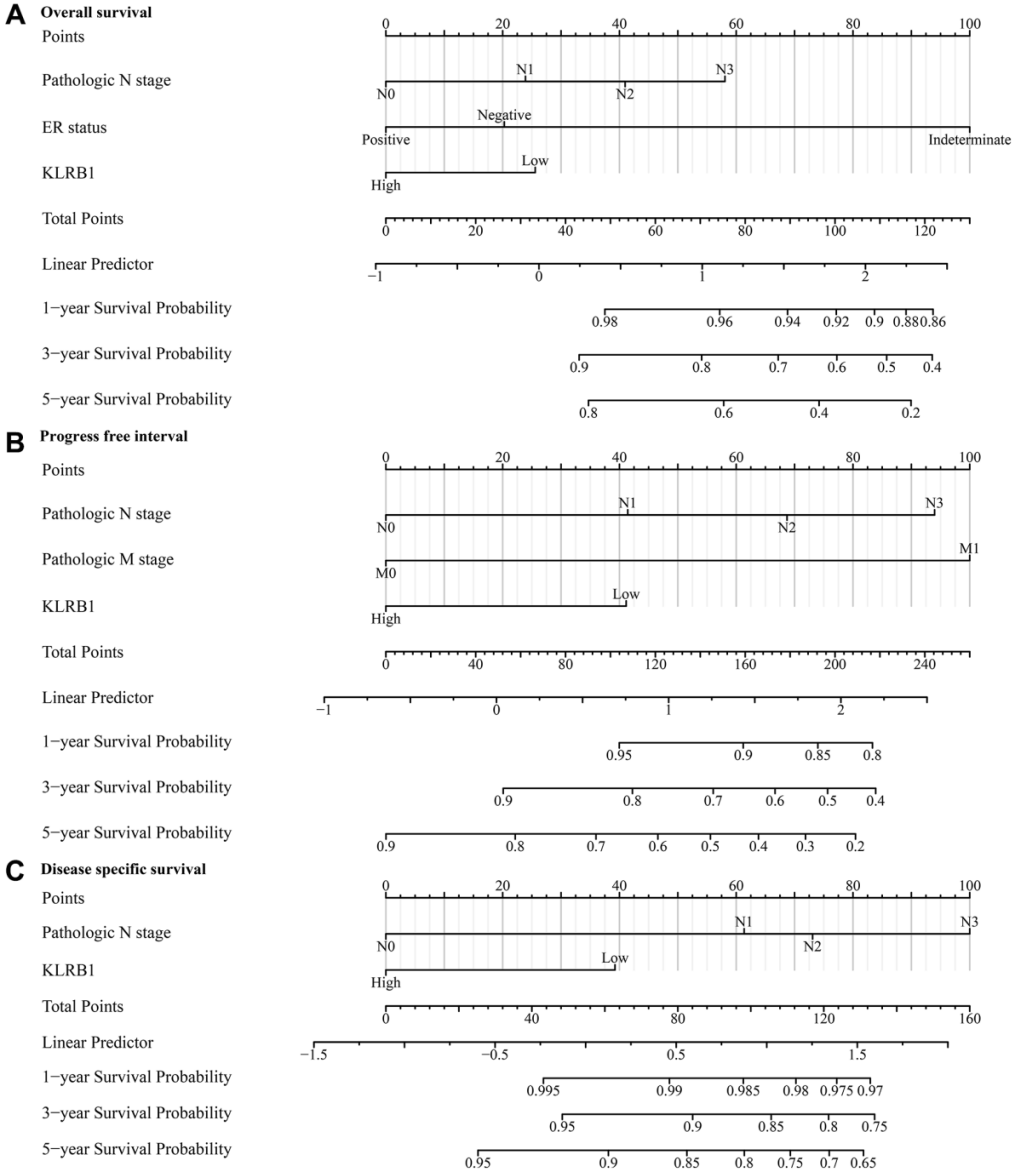
**KLRB1-associated prognostic nomograms in BRCA patients.** (**A**) OS; (**B**) PFI; (**C**) DSS. Abbreviations: BRCA: breast invasive carcinoma; OS: overall survival; PFI: progress free interval; DSS: disease specific survival.

Univariate COX regression analysis demonstrated that M stage, ER status, N stage, PAM50 status, T stage, and decreased KLRB1 level were adverse factors affecting the survival of BRCA patients (Table 2). Multivariate COX regression analysis demonstrated that N stage, ER status (positive), and decreased KLRB1 level were independent risk factors for survival in BRCA patients (Table 2). Univariate COX regression analysis demonstrated that M stage, PR status, T stage, ER status, N stage, PAM50 status and decreased KLRB1 levels were adverse factors affecting DSS in BRCA patients (Table 3). Multivariate COX regression analysis demonstrated that N stage and decreased KLRB1 level were independent risk factors for DSS in BRCA patients (Table 3). Univariate COX regression analysis demonstrated that T stage, PR status, M stage, ER status, N stage, and decreased KLRB1 expression were adverse factors affecting PFI in BRCA patients (Table 4). Multivariate COX regression analysis demonstrated that N stage, decreased KLRB1 level, and M stage were independent risk factors for PFI in BRCA patients (Table 4). The relationship between KLRB1 levels and survival was further analyzed by constructing KLRB1-related prognostic nomograms for BRCA patients based on these results of COX methods (Figure 3).

**Table 2:** OS-related factors in BRCA.

**Characteristics**	**Total (*N*)**	**HR (95% CI)**	***P*-value**	**HR (95% CI)**	***P*-value**
T stage	1083		0.004		
T1	277	Reference		Reference	
T2	631	1.336 (0.890–2.006)	0.162	0.928 (0.576–1.494)	0.758
T3	140	1.551 (0.921–2.612)	0.099	1.091 (0.580–2.052)	0.787
T4	35	3.759 (1.959–7.213)	<0.001	1.902 (0.877–4.124)	0.104
N stage	1067		<0.001		
N0	516	Reference		Reference	
N1	358	1.947 (1.322–2.865)	<0.001	1.591 (1.023–2.476)	0.039
N2	116	2.522 (1.484–4.287)	<0.001	2.888 (1.583–5.269)	<0.001
N3	77	4.191 (2.318–7.580)	<0.001	3.779 (1.714–8.329)	<0.001
M stage	925		<0.001		
M0	905	Reference		Reference	
M1	20	4.266 (2.474–7.354)	<0.001	1.475 (0.726–2.998)	0.282
PR status	1037		0.191		
Negative	342	Reference			
Indeterminate	4	0.825 (0.113–6.028)	0.850		
Positive	691	0.729 (0.521–1.020)	0.065		
ER status	1038		0.005		
Negative	240	Reference		Reference	
Indeterminate	2	13.090 (3.128–54.779)	<0.001	7.193 (0.892–58.002)	0.064
Positive	796	0.709 (0.493–1.019)	0.063	0.521 (0.290–0.933)	0.028
HER2 status	729		0.095		
Negative	560	Reference			
Indeterminate	12	0.000 (0.000–Inf)	0.994		
Positive	157	1.593 (0.973–2.609)	0.064		
PAM50	1086		0.024		
Normal	40	Reference		Reference	
LumA	563	0.659 (0.302–1.438)	0.295	0.928 (0.326–2.636)	0.888
LumB	206	1.101 (0.486–2.495)	0.818	1.187 (0.404–3.490)	0.755
Her2	82	1.498 (0.621–3.614)	0.369	1.253 (0.385–4.073)	0.708
Basal	195	0.847 (0.371–1.935)	0.694	0.947 (0.303–2.958)	0.925
KLRB1	1086		<0.001		
Low	543	Reference		Reference	
High	543	0.550 (0.396–0.763)	<0.001	0.533 (0.361–0.787)	0.002

Abbreviations: BRCA: breast invasive carcinoma; OS: overall survival.

**Table 3:** DSS-related factors in BRCA.

**Characteristics**	**Total (*N*)**	**HR (95% CI)**	***P*-value**	**HR (95% CI)**	***P*-value**
T stage	1063		<0.001		
T1	275	Reference		Reference	
T2	620	1.555 (0.880–2.748)	0.129	1.020 (0.528–1.968)	0.954
T3	134	1.746 (0.838–3.637)	0.137	0.955 (0.379–2.406)	0.922
T4	34	6.713 (3.005–14.992)	<0.001	2.462 (0.932–6.505)	0.069
N stage	1048		<0.001		
N0	513	Reference		Reference	
N1	348	3.376 (1.918–5.942)	<0.001	2.496 (1.333–4.673)	0.004
N2	112	3.758 (1.758–8.032)	<0.001	4.589 (1.994–10.560)	<0.001
N3	75	7.132 (3.321–15.316)	<0.001	5.613 (1.957–16.095)	0.001
M stage	906		<0.001		
M0	887	Reference		Reference	
M1	19	7.475 (3.999–13.972)	<0.001	2.102 (0.912–4.846)	0.081
PR status	1018		0.013		
Negative	334	Reference		Reference	
Indeterminate	4	1.337 (0.180–9.927)	0.777	3.011 (0.357–25.408)	0.311
Positive	680	0.517 (0.333–0.805)	0.003	0.741 (0.302–1.821)	0.514
ER status	1019		0.013		
Negative	232	Reference		Reference	
Indeterminate	2	7.769 (1.045–57.757)	0.045	0.000 (0.000–Inf)	0.996
Positive	785	0.557 (0.349–0.888)	0.014	0.400 (0.158–1.010)	0.053
HER2 status	718		0.360		
Negative	552	Reference			
Indeterminate	12	0.000 (0.000–Inf)	0.997		
Positive	154	1.477 (0.740–2.948)	0.269		
PAM50	1066		0.026		
Normal	39	Reference		Reference	
LumA	556	1.232 (0.296–5.133)	0.775	1.923 (0.253–14.593)	0.527
LumB	201	2.071 (0.478–8.966)	0.330	1.993 (0.254–15.638)	0.512
Her2	80	3.432 (0.760–15.490)	0.109	1.803 (0.210–15.501)	0.591
Basal	190	2.241 (0.524–9.577)	0.276	1.662 (0.202–13.687)	0.637
KLRB1	1066		0.002		
Low	532	Reference		Reference	
High	534	0.509 (0.328–0.791)	0.003	0.506 (0.298–0.857)	0.011

Abbreviations: BRCA: breast invasive carcinoma; DSS: disease specific survival.

**Table 4:** PFI–related factors in BRCA.

**Characteristics**	**Total (*N*)**	**HR (95% CI)**	***P*-value**	**HR (95% CI)**	***P*-value**
T stage	1083		<0.001		
T1	277	Reference		Reference	
T2	631	1.618 (1.045–2.507)	0.031	1.245 (0.756–2.050)	0.389
T3	140	2.184 (1.273–3.747)	0.005	1.360 (0.699–2.647)	0.365
T4	35	6.272 (3.268–12.034)	<0.001	2.274 (0.975–5.303)	0.057
N stage	1067		<0.001		
N0	516	Reference		Reference	
N1	358	1.973 (1.326–2.936)	<0.001	1.638 (1.047–2.561)	0.031
N2	116	2.485 (1.444–4.279)	0.001	2.748 (1.505–5.018)	<0.001
N3	77	4.959 (2.832–8.684)	<0.001	3.569 (1.606–7.931)	0.002
M stage	925		<0.001		
M0	905	Reference		Reference	
M1	20	8.345 (4.847–14.368)	<0.001	2.972 (1.370–6.448)	0.006
PR status	1037		0.003		
Negative	342	Reference		Reference	
Indeterminate	4	0.789 (0.107–5.817)	0.816	0.995 (0.128–7.757)	0.996
Positive	691	0.556 (0.399–0.776)	<0.001	0.579 (0.333–1.008)	0.053
ER status	1038		0.015		
Negative	240	Reference		Reference	
Indeterminate	2	4.156 (0.570–30.286)	0.160	0.000 (0.000–Inf)	0.994
Positive	796	0.619 (0.434–0.883)	0.008	0.578 (0.324–1.030)	0.063
HER2 status	729		0.759		
Negative	560	Reference			
Indeterminate	12	1.213 (0.167–8.796)	0.849		
Positive	157	1.230 (0.713–2.122)	0.458		
PAM50	1086		0.080		
Normal	40	Reference			
LumA	563	0.722 (0.331–1.574)	0.412		
LumB	206	0.871 (0.377–2.015)	0.747		
Her2	82	1.536 (0.632–3.738)	0.344		
Basal	195	1.081 (0.478–2.445)	0.852		
KLRB1	1086		<0.001		
Low	543	Reference		Reference	
High	543	0.570 (0.410–0.793)	<0.001	0.496 (0.334–0.735)	<0.001

Abbreviations: BRCA: breast invasive carcinoma; PFI: progress free interval.

### Biological functions and mechanisms involved in KLRB1

**Figure 4:**
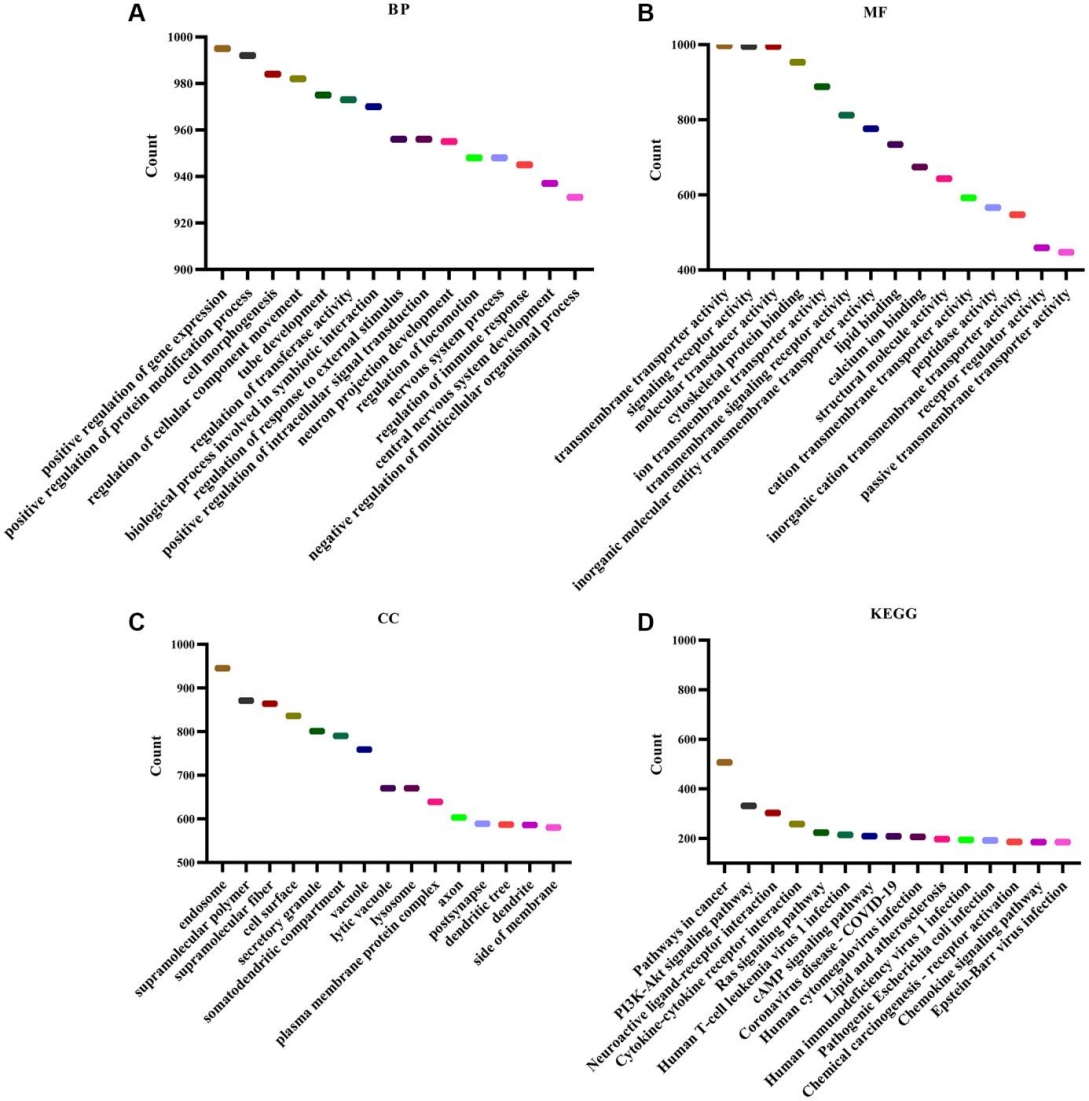
**Functions and signaling mechanisms involved in KLRB1.** (**A**) BP; (**B**) MF; (**C**) CC; (**D**) KEGG.

GSEA analysis revealed that KLRB1 was involved in various biological functions and pathways related to immune response, and cancer (Figure 4A–4C). These functions included natural killer cell proliferation, dendritic cell apoptotic process, immune response, B cell mediated immunity, memory T cell differentiation, regulation of T cell tolerance induction, negative regulation of natural killer cell mediated immunity, dendritic cell migration, regulation of B cell activation, positive regulation of B cell differentiation, regulation of natural killer cell mediated immunity, negative regulation of B cell proliferation, regulation of natural killer cell mediated cytotoxicity, regulatory T cell, T-helper 1 and T-helper 17 cell differentiation, regulation of T cell mediated cytotoxicity, negative regulation of T cell mediated immunity, and others (Figure 4A–4C). The pathways regulated included T cell receptor, NF-κB, B cell receptor, chemokine, PD-L1 expression in cancer and PD-1 checkpoint, Toll-like receptor, TNF, JAK-STAT, NOD-like receptor, IL-17, apoptosis, PI3K-Akt, and other pathways (Figure 4D).

### Elevated levels of KLRB1 were found to effectively retard cell growth and migration in BRCA

**Figure 5:**
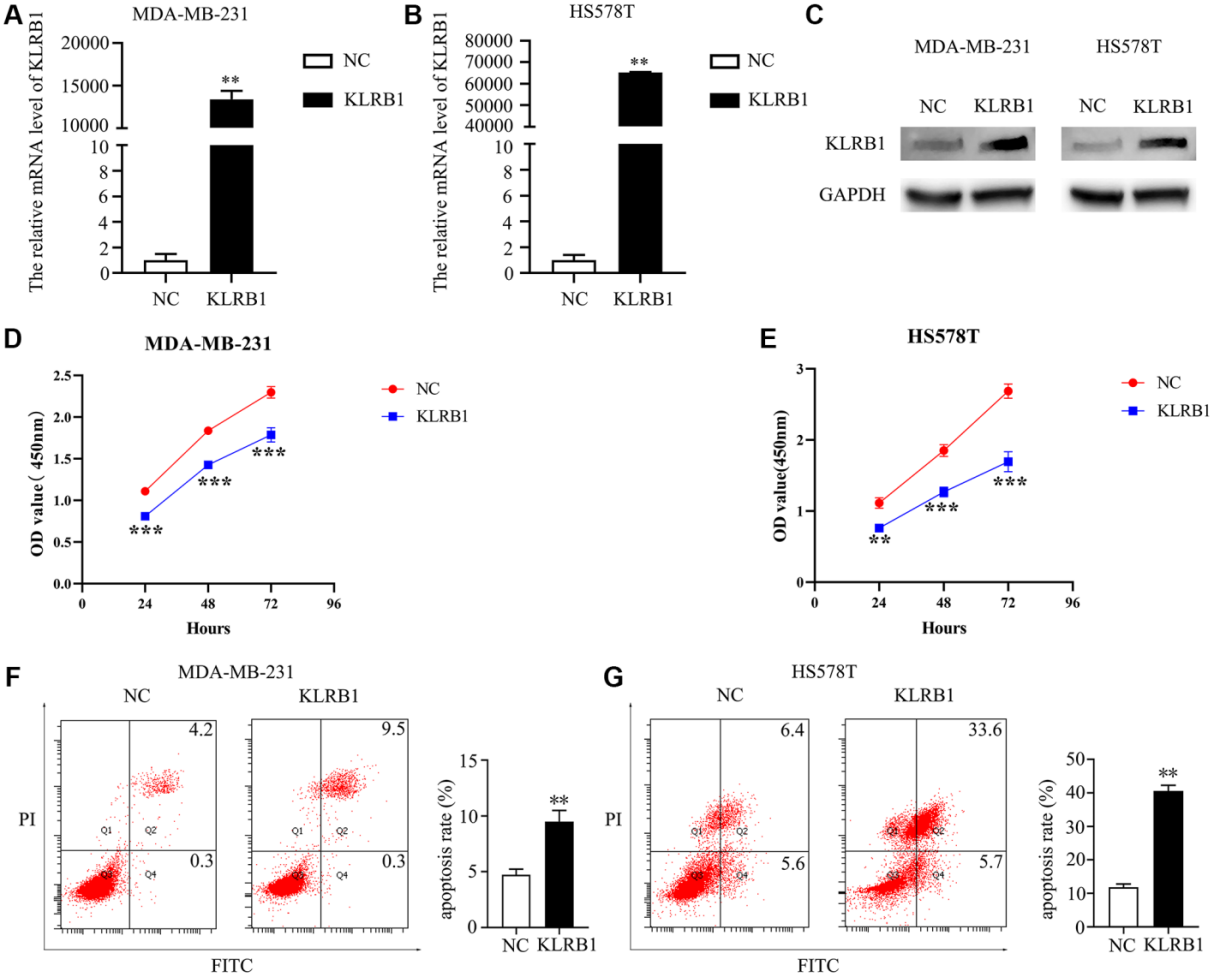
**Overexpression of KLRB1 inhibited proliferation and promoted apoptosis of breast cancer cells.** (**A**–**C**) Cell models on KLRB1 overexpression; (**D**) Cell viability of MDA-MB-231; (**E**) Cell viability of HS578T; (**F**, **G**) Apoptosis of MDA-MB-231 and HS578T cells.

**Figure 6:**
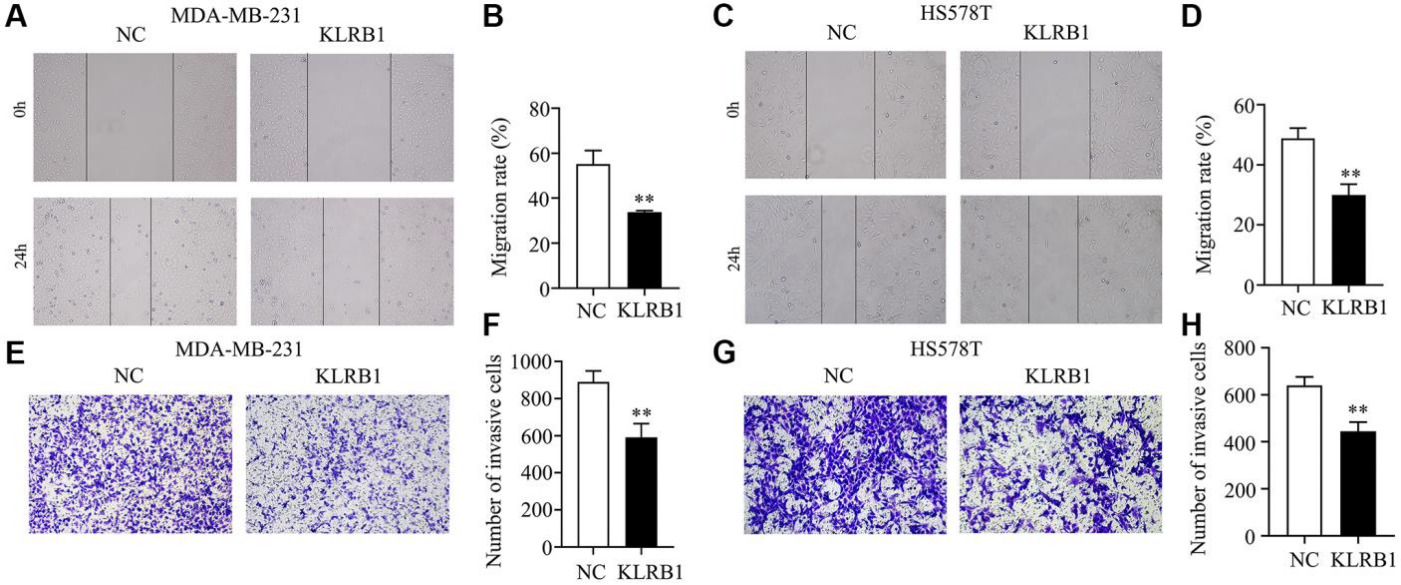
**Overexpression of KLRB1 inhibited migration and invasion of breast cancer cells.** (**A**–**D**) The migration of MDA-MB-231 and HS578T cells; (**E**–**H**) The invasion of MDA-MB-231 and HS578T cells.

Significantly elevated KLRB1 levels were observed in the KLRB1 overexpression group of MDA-MB-231 and HS578T cells (Figure 5A–5C). Moreover, the CCK-8 assay indicated that increased KLRB1 levels significantly inhibited the proliferation of MDA-MB-231 and HS578T cells, with a statistically significant difference observed at 48 h and 72 h (Figure 5D, 5E). Overexpression of KLRB1 also facilitated apoptosis in BRCA cells, as confirmed by flow cytometry analysis (Figure 5F, 5G). Furthermore, the results of wound healing and transwell methods revealed that KLRB1 overexpression inhibited cancer cell migration and invasiveness (Figure 6).

### Decreased expression of KLRB1 was found to be significantly associated with alterations in the BRCA immune microenvironment

In this study, we utilized cancer tissues from the TCGA database to investigate the correlation between KLRB1 expression levels and various immune parameters. Pearson correlation analysis showed that reduced KLRB1 expression levels were significantly positively correlated with stromal score (r = 0.464), immune score (r = 0.786), and estimate score (r = 0.725) levels. We also observed that reduced KLRB1 levels were positively correlated with the T cells, cytotoxic cells, B cells, dendritic cells, Th1 cells, iDC, TFH, pDC, CD8 T cells, aDC, NK CD56dim cells, TReg, Tem, T helper cells, macrophages, neutrophils, Tcm, mast cells, eosinophils, NK CD56bright cells, and NK cells, and negatively correlated with Th17 cells, Th2 cells, and Tgd cells (Table 5). Moreover, using the TIMER database, we found that decreased KLRB1 levels were associated with BRCA purity and immune cell infiltration (Table 6). Specifically, this correlation was observed in patients with BRCA and its subtypes (basal, HER2, and luminal), where reduced KLRB1 expression levels were linked to tumor purity, B cells, CD8^+^ T cells, CD4^+^ T cells, neutrophils, and dendritic cells (Table 6).

**Table 5:** KLRB1 was correlated with immune cells in BRCA.

**Genes**	**Immune cells**	**Cor**	***P*-value**
KLRB1	T cells	0.800555814	2.5381E-249
KLRB1	Cytotoxic cells	0.772704052	1.5661E-221
KLRB1	B cells	0.726905842	1.554E-183
KLRB1	DC	0.624347399	2.5641E-121
KLRB1	Th1 cells	0.567627503	6.31938E-96
KLRB1	iDC	0.563483503	2.9273E-94
KLRB1	TFH	0.556155421	2.26529E-91
KLRB1	pDC	0.546873111	8.17311E-88
KLRB1	CD8 T cells	0.544498333	6.37781E-87
KLRB1	aDC	0.538472388	1.08898E-84
KLRB1	NK CD56dim cells	0.533117402	9.62049E-83
KLRB1	TReg	0.502526013	2.86579E-72
KLRB1	Tem	0.488406716	8.78159E-68
KLRB1	T helper cells	0.434277185	2.09213E-52
KLRB1	Macrophages	0.372023474	7.37767E-38
KLRB1	Neutrophils	0.348801477	3.44797E-33
KLRB1	Tcm	0.328567293	1.98905E-29
KLRB1	Mast cells	0.263560855	3.83904E-19
KLRB1	Eosinophils	0.110586626	0.000218549
KLRB1	NK CD56bright cells	0.101864544	0.00066551
KLRB1	NK cells	0.075209353	0.012078438
KLRB1	Th17 cells	-0.06323577	0.034910869
KLRB1	Th2 cells	-0.077270616	0.009913404
KLRB1	Tgd	-0.097763283	0.001091917

Abbreviation: BRCA: breast invasive carcinoma.

**Table 6:** KLRB1 was positively correlated with immune cells in BRCA and subtypes of BRCA.

**Cancer**	**Variable**	**Coefficient**	***P***	**Cancer**	**Variable**	**Coefficient**	***P***
BRCA	Purity	−0.57993546	1.74E-90	BRCA-Her2	Purity	−0.560030444	3.99E-06
BRCA	B Cell	0.381839069	2.87E-35	BRCA-Her2	B Cell	0.343013091	0.008390474
BRCA	CD8^+^ T Cell	0.581726722	1.86E-89	BRCA-Her2	CD8^+^ T Cell	0.698971526	1.48E-09
BRCA	CD4^+^ T Cell	0.588336596	1.18E-90	BRCA-Her2	CD4^+^ T Cell	0.666070012	1.16E-08
BRCA	Macrophage	0.162186587	3.18E-07	BRCA-Her2	Macrophage	−0.037896091	0.777616237
BRCA	Neutrophil	0.466897405	1.18E-52	BRCA-Her2	Neutrophil	0.564917261	3.85E-06
BRCA	Dendritic Cell	0.566350098	9.05E-82	BRCA-Her2	Dendritic Cell	0.627201232	2.31E-07
BRCA-Basal	Purity	−0.672702913	2.53E-18	BRCA-Luminal	Purity	−0.538017747	2.71E-42
BRCA-Basal	B Cell	0.541304362	8.52E-11	BRCA-Luminal	B Cell	0.396850086	7.51E-22
BRCA-Basal	CD8^+^ T Cell	0.491754727	7.65E-09	BRCA-Luminal	CD8^+^ T Cell	0.649429691	1.22E-65
BRCA-Basal	CD4^+^ T Cell	0.416663954	1.81E-06	BRCA-Luminal	CD4^+^ T Cell	0.610932333	3.78E-56
BRCA-Basal	Macrophage	0.066792053	0.455607245	BRCA-Luminal	Macrophage	0.184699084	1.51E-05
BRCA-Basal	Neutrophil	0.401642293	1.37E-05	BRCA-Luminal	Neutrophil	0.44718469	1.29E-27
BRCA-Basal	Dendritic Cell	0.507719351	8.13E-09	BRCA-Luminal	Dendritic Cell	0.56606425	1.19E-46

### The KLRB1 expression was found to be correlated with immune cell markers and immunotherapy in BRCA

**Figure 7:**
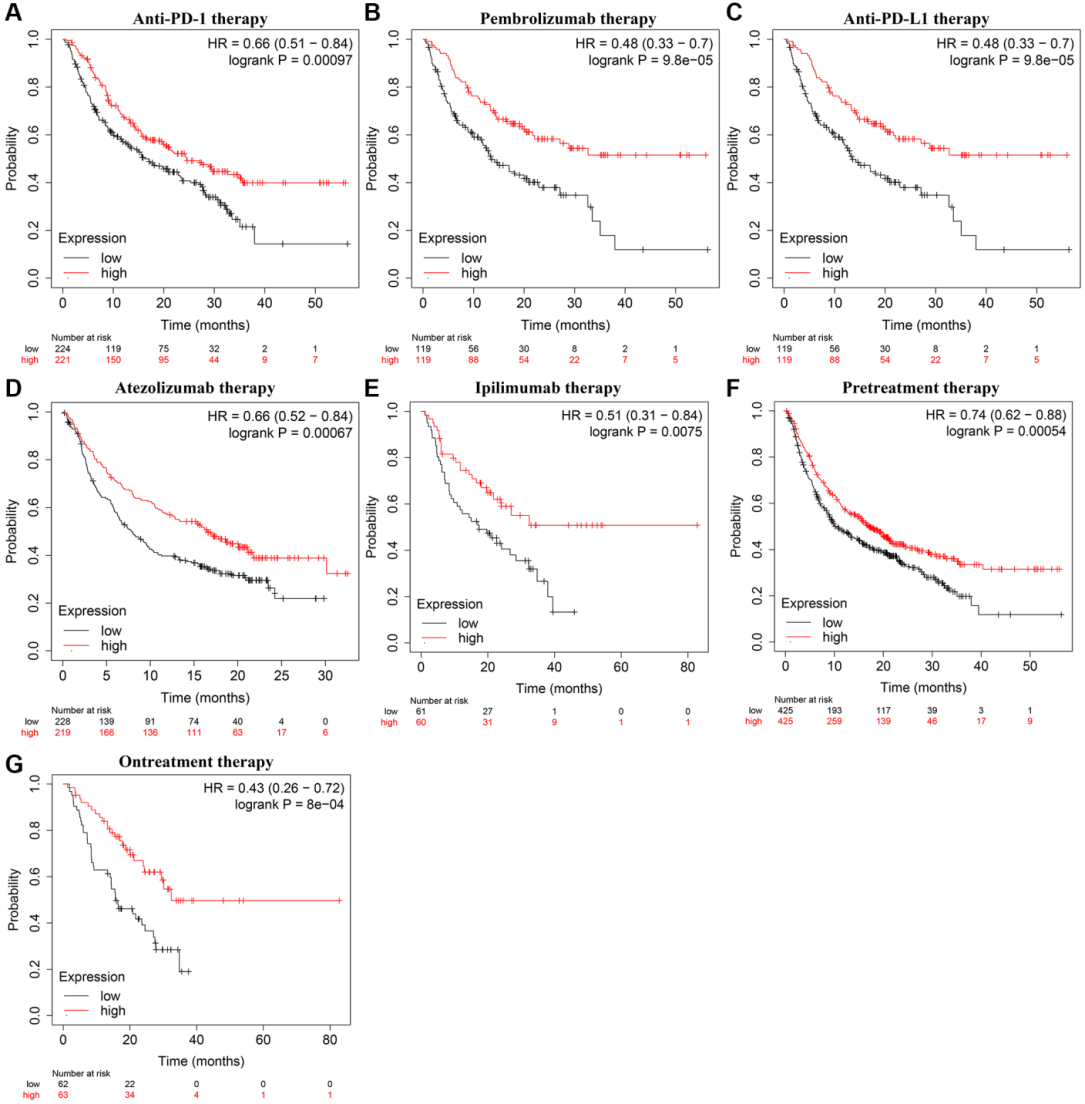
**KLRB1 overexpression was correlated with the OS in cancer patients on immunotherapy.** (**A**) Anti-PD-1; (**B**) Pembrolizumab; (**C**) Anti-PD-L1; (**D**) Atezolizumab; (**E**) Ipilimumab; (**F**) Pretreatment; (**G**) Ontreatment. Abbreviation: OS: overall survival.

The TIMER database analysis illustrated a positive correlation between KLRB1 expression and immune-cell markers such as PDCD1 (r = 0.731), CD274 (r = 0.438), and CTLA4 (r = 0.648). Moreover, KLRB1 expression exhibited a positive correlation with the expression of PDCD1 (r = 0.624), CD274 (r = 0.317), and CTLA4 (r = 0.541) under condition of tumor purity, and PDCD1 (r = 0.731), CD274 (r = 0.441), and CTLA4 (r = 0.643) under condition of age in BRCA. We also observed positive correlations between KLRB1 expression and PDCD1 (r = 0.759, 0.744, and 0.846), CD274 (r = 0.599, 0.499, and 0.48), and CTLA4 (r = 0.784, 0.61, and 0.812) levels in BRCA subtypes basal, luminal, and HER2, respectively. Using the GEPIA database, we discovered similar positive correlations between KLRB1 expression and PDCD1, CD274, and CTLA4 levels in normal breast and BRCA tissues as presented in Supplementary Figure 1. Additionally, In the cancer patients treated with anti-PDCD1 (Pembrolizumab), anti-CD274 (Atezolizumab), or anti-CTLA4 (Ipilimumab) of the K-M plotter database, elevated KLRB1 expression exhibited better OS and progression-free survival (PFS) after grouping with KLRB1 expression median value (Figure 7 and Supplementary Figure 2A–2F). These findings suggest that KLRB1 plays a vital role in immune escape in the BRCA microenvironment.

## DISCUSSION

BC remains a significant cause of morbidity and mortality among patients [16]. Consequently, the development of tumor molecular biology has led to the emergence of targeted molecular therapies that may improve the prognosis of cancer patients [17–19]. For instance, Secreted Protein Acidic and Rich in Cysteine (SPARC) is distinctly expressed in various cells, including tumor cells, cancer-associated fibroblasts (CAFs), tumor-associated macrophages, endothelial cells, and tumor-infiltrating lymphocytes [19]. SPARC overexpression has been identified as a risk factor for reduced recurrence-free survival of CAF-cancer patients. Moreover, SPARC secreted by CAFs promotes tumor growth by inhibiting adhesion of triple-negative BC cells whilst stimulating invasion and metastasis [19]. Recently, KLRB1 has become an increasing focus in tumor research as it has been associated with tumor progression [11–15, 20]. Xu and Huang et al. found that KLRB1 is a tumor suppressor gene involved in the BC progression. We conducted in-depth research and find that KLRB1 expression has important clinical value in BRCA patients. Specifically, KLRB1 expression was significantly decreased, and the area under the curve of KLRB1 was 0.7125 in BRCA, indicating its potential as a diagnostic biomarker. In BRCA patients, decreased KLRB1 expression was associated with poorer OS, DSS, PFI, DMFS, RFS, M stage, pathological stage, age, and survival status. Furthermore, Cox regression analysis showed that KLRB1 downregulation was an independent risk factor for poor prognosis among BRCA patients and was associated with advanced disease stages and worse clinical outcomes. Therefore, KLRB1 expression appears promising as a biomarker for assessing the prognosis of BRCA patients and may serve as a potential therapeutic target.

Recent literature has indicated gene involvement in the progression of BRCA, as evident from multiple studies [8–10, 21, 22]. For example, the StAR related lipid transfer domain containing 4 (STARD4) was found to be highly expressed in BRCA and its overexpression was associated with a poor prognosis [10]. Inhibition of STARD4 expression could suppress proliferation and migration and promote apoptosis of BRCA cells. Another gene of interest, KLRB1, was identified through GSEA analysis and basic studies as having an important function in BRCA progression. GSEA analysis demonstrated KLRB1’ involvement in cell proliferation, immune response, cell differentiation, and cell migration. Cell studies confirmed that increased KLRB1 expression could inhibit BRCA cell proliferation and migration while promoting cell apoptosis. Additionally, KLRB1 expression was observed to involve the cancer pathways (such as the NF-κB, PI3K-Akt, and TNF pathways). However, further studies are needed to confirm the specific roles of KLRB1 in these mechanisms. The available literature has established a close link between the cancer mechanism and the progression of BRCA [23–25]. Overall, our results suggest that KLRB1 acts as a tumor suppressor gene in BRCA, consistent with database analysis.

Immunotherapy is a promising treatment that can improve the quality of life and survival time of cancer patients [26–28]. For example, Zhang et al. demonstrated that combining PDCD1/CD274 inhibitors with chemotherapy improved pCR, EFS, and OS in triple-negative breast cancer. Similarly, the PFS of patients with advanced triple-negative breast cancer treated with PDCD1/CD274 inhibitors combined with chemotherapy was longer than those treated with chemotherapy alone [27]. In this study, we found that decreased KLRB1 levels were associated with lower stromal score, immune score, and tumor purity, as well as lower levels of immune cells such as T cells, cytotoxic cells, B cells, dendritic cells, and natural killer cells. Additionally, decreased KLRB1 levels were associated with decreased levels of CD8^+^ T cells, CD4^+^ T cells, neutrophils, and dendritic cells in patients with BRCA and its subtypes basal, HER2, and luminal. Furthermore, KLRB1 expression was positively correlated with immune cell markers such as PDCD1, CD274, and CTLA4. K-M survival analysis indicated that OS and PFS were better in cancer patients treated with PDCD1, CD274, and CTLA4 with elevated KLRB1 expression. In addition, GSEA results showed that KLRB1 was significantly associated with PD-L1 expression and PD-1 checkpoint pathway in cancer. These findings suggest that KLRB1 plays an important role in the progression of BRCA using PD-1 and PD-L1 and may have significant clinical value in BRCA immunotherapy.

This study aimed to evaluate the biological roles of KLRB1 in BRCA through database analysis and basic research, providing a new candidate molecule for treating BRCA patients with positive clinical value. However, further research is needed to confirm the relationship between KLRB1 expression and clinical features, cancer subtypes, and prognosis using clinical tissue samples of BRCA patients, and explore the signaling mechanisms of KLRB1 in BRCA progression in the future. In brief, our results reveal that KLRB1 levels are significantly lower, and decreased KLRB1 expression correlates with age, T stage, pathological stage, HER2 positivity, and poor prognosis in BRCA. Therefore, reduced KLRB1 expression is a potential biomarker for poor prognosis in BRCA patients. Conversely, increased KLRB1 expression could reduce BRCA cell proliferation, migration, and invasion, while promoting apoptosis. Furthermore, KLRB1 expression levels are significantly correlated with tumor purity, immune infiltration, and immunotherapy-related markers such as PDCD1, CD274, and CTLA4. Therefore, KLRB1 may have clinical implications in predicting the prognosis of BRCA patients.

## CONCLUSION

Decreased KLRB1 expression in BRCA is associated with poor prognosis and immune microenvironment. This study also highlights KLRB1 as a potential molecular marker for poor prognosis in BRCA patients, and therefore, it may provide clinical implications for the management of patients with BRCA.

## MATERIALS AND METHODS

### Identification of KLRB1 expression levels in BRCA

The transcriptome data of FPKM patients were downloaded from TCGA database official website. Among them, there were 113 cases of normal breast tissue and 1113 cases of BRCA tissue in TCGA database. 113 cases of normal breast tissues and 113 cases of BRCA tissues were paired, and then the data were combined and sorted out to identify the expression levels of KLRB1 in normal breast tissues and BRCA tissues by using the differential analysis. The transcriptome data of patients with FPKM were obtained from the official website of TCGA database. The database contained 113 cases of normal breast tissue and 1113 cases of BRCA tissue. We paired 113 cases of normal breast tissues with 113 cases of BRCA tissues by combining and sorting out the data to determine the expression levels of KLRB1 in normal breast and BRCA tissues through differential analysis methods.

### The relationship between KLRB1 expression and clinical characteristics of BRCA patients

Clinical data for a total of BRCA patients were obtained from the TCGA database. We also grouped the clinical characteristics (Such as the T stage, M stage, pathological stage, N stage, age, ER status, PR status, HER2 status, and menopause status) of BRCA patients to determine the statistical significance of KLRB1 levels within each group, with *P* < 0.05 being considered statistically significant.

### Diagnostic and prognostic values of KLRB1 in BRCA

ROC curves are commonly used to assess the diagnostic value of genes [29–31]. Thus, we utilized ROC analysis to determine the diagnostic value of KLRB1 in normal and BRCA tissues obtained from the TCGA database. The K-M plotter database, which covers sequencing data from the TCGA and Gene Expression Omnibus (GEO) databases, was used to determine the relationship between KLRB1 levels and prognosis in BRCA patients. This database was also used to evaluate the relationship between KLRB1 levels and clinicopathological features influencing the prognosis of BRCA patients. Moreover, the immunotherapy module in the K-M plotter database was used to determine the relationship between KLRB1 levels and the OS and PFS of patients with cancer treated through immunotherapy. Additionally, we downloaded clinical data for BRCA patients from the TCGA database website, then aggregated the KLRB1 expression data and prognosis data to identify the clinical value of KLRB1 expression in the prognosis of BRCA patients through K-M survival analysis.

### Construction of KLRB1 related-prognostic nomograms

We employed univariate COX regression analysis to assess the relationship between HER2 status, T stage, M stage, PR status, N stage, ER status, PAM50 status, KLRB1 levels, and OS, DSS, and PFI of cancer patients based on data from the TCGA database. A significance threshold of *P* < 0.05 was used for inclusion in the multivariate COX regression analysis. Following the multivariate COX regression analysis, results were filtered using *P* < 0.05, and KLRB1-related nomograms were constructed based on the multivariate COX regression analysis results.

### TIMER database

The TIMER database provides the means to analyze the relationship between genes and tumor purity as well as immune cell infiltration in BRCA and its subtypes. Specifically, we utilized the gene module in the TIMER database to examine the association between KLRB1 levels and immune cell levels, as well as tumor purity. Furthermore, the correlation analysis was used to investigate the relationship between KLRB1 levels and BRCA immune cell markers, with *P* < 0.05 being used as the significance threshold.

### GEPIA database

The GEPIA database is an online analytical tool that utilizes data from both the TCGA and Genotype-Tissue Expression (GTEx) databases. To confirm the relationship between KLRB1 levels and BRCA immune cell markers, we utilized the correlation analysis module data available on the GEPIA database, with a statistical significance value of *P* < 0.05.

### Function and signaling mechanisms of KLRB1

We utilized the GSEA analysis method to investigate the biological functions and pathways involved in a gene [29–31]. In this study, we selected gene expression data from BRCA patients in the TCGA database, and then used the GSEA module of the CAMOIP database to explore the signaling mechanisms associated with KLRB1 expression levels during BRCA progression. A corrected *P*-value of less than 0.05 was used as the significance threshold.

### Cell culture and construction of cell models of KLRB1 overexpression

MDA-MB-231 and HS578T cells were cultured with RPMI-1640 (Thermo Fisher Scientific, USA) basal medium supplemented with 10% fetal bovine serum. BC cells were maintained at 37°C and passaged every 3–4 days. The pCDNA3.1 expression vector was synthesized by GeneCopoeia (China). MDA-MB-231 and HS578T cells were cultured in 6-well plates at appropriate densities for 24 hours, following which KLRB1 overexpression and control vectors were respectively introduced using Lipofectamine 3000 (Thermo Fisher Scientific, USA). RNA and protein were collected 24 hours after transfection, and KLRB1 expression levels were confirmed in MDA-MB-231 and HS578T cells using RT-PCR and Western blotting.

### qRT-PCR

For RNA extraction, Trizol solution was added to 6-well plates containing MDA-MB-231 and HS578T cells, after which the BC cells were lysed using a pipetting gun and transferred to a new centrifuge tube. Standard procedures were applied, and this involved the addition of chloroform, isopropanol, 75% ethanol and DEPC treated water. The RNA concentration was determined by calculating the absorbance ratio detected with a spectrophotometer. Reverse transcription was performed according to the instructions provided in the Takara kit, and PCR amplification was conducted to synthesize KLRB1 cDNA. GAPDH was utilized as the internal control. The KLRB1 primers used were ATCTCTTCCTCGGGATGTCTGTCAG (Forward primer) and AGGATGT CACTGAAACACTCAACCC (Reverse primer).

### Western blotting

A cell lysis solution was added to MDA-MB-231 and HS578T cells in 6-well plates, with the lysate being completely mixed with the cancer cells using a pipetting gun and subsequently transferred to a new centrifuge tube. Protein concentrations were calculated for cells via the BCA method before proceeding with standard electrophoresis, electroporation, primary antibody incubation, secondary antibody incubation, and exposure sequences. The relative expression levels of KLRB1 in control and KLRB1 overexpression cells were subsequently calculated. Protein concentrations for primary antibodies, including GAPDH (Always, China) and KLRB1 (Abclonal, China), were maintained at 1:3000 and 1:5000, respectively, during incubation.

### Cell proliferation

The CCK-8 assay is a common method for assessing the proliferation ability of cancer cells. In this study, MDA-MB-231 and HS578T cells, which had demonstrated good growth after cell transfection, were counted and seeded at a density of 3000 cells/well in 96-well plates. The proliferation ability of these cell lines was evaluated as per the manufacturer’s instructions for the CCK-8 kit. Briefly, a CCK-8 solution of 10 ul/well was added at 24, 48, and 72 hours, and the cells were then incubated at 37°C and 5% CO_2_ for additional 2 hours. The absorbance values of MDA-MB-231 and HS578T cells were determined using a spectrophotometer (Bio-Rad, USA), and statistical analysis was performed to compare the control and KLRB1 overexpression groups. The experiments were performed in triplicate.

### Cell apoptosis

Following transfection, MDA-MB-231 and HS578T cells in good growth condition were collected using trypsin without ethylenediaminetetraacetic acid. The collected cells were centrifuged and washed once with phosphate buffer, before being stained with 500 μl of buffer, 1 μl of Annexin V-PE, and 5 μl of 7-AAD (Kaiji, Nanjing, China). The effects of KLRB1 overexpression on the apoptosis of MDA-MB-231 and HS578T cells were evaluated using flow cytometry, under dark conditions. The experiment was conducted three times.

### Cell migration and invasion

When MDA-MB-231 and HS578T cells in good growth condition after transfection had reached 80–90%, a 0.2 ml gun tip was used to create a straight line along the bottom of the 6-well plate, after which the cells were washed with a phosphate buffer solution. Control and KLRB1 overexpressing BC cells were then photographed and labeled using microscopy at this point, as well as at 24 h in the future. The experiment was replicated three times to ensure accuracy. Transwell assays were performed on MDA-MB-231 and HS578T cells after transfection. Basolateral chambers were balanced with 600 μL of DMEM containing 10% FBS, 200 μL of MDA-MB-231 cell or HS578T cell suspension in FBS-free DMEM were cultivated into the apical chambers. Culturing for 24 h, the transwell chambers were fixed with 4% paraformaldehyde for 10 minutes, stained with 0.1% crystal violet for 15 minutes, and washed with PBS for three times. An inverted fluorescence microscope was used to observe the transmembrane cells.

### Statistical analysis

To determine the expression levels of the KLRB1 gene in BRCA tissues, the Wilcoxon rank sum test was employed. Moreover, the prognostic and diagnostic values of the KLRB1 gene were analyzed through survival analysis and ROC analysis. Differences in cell proliferation, apoptosis, and migration ability between the control group and KLRB1 overexpression group were determined using the *t*-test. Pearson correlation analysis was applied to investigate the relationship between KLRB1 gene and BRCA immune cell infiltration. The figure was displayed through GraphPad Prism and R languages, and a statistical significance level of *P* < 0.05 was considered as the criterion for determining significance.

### Data availability

The data of TCGA, K-M plotter, GTEx, Xiantao Academic, and GEO databases were obtained or by contacting the corresponding authors.

## Supplementary Materials

Supplementary Figures
