# Supplementary material for: Inhibiting KLRB1 expression is associated with impairing cancer immunity and leading to cancer progression and poor prognosis in breast invasive carcinoma patients
Source: Aging (Albany NY). 2023 Nov 20;15(22):13265–86. doi: 10.18632/aging.205239 (PMC10713394; doi:10.18632/aging.205239)
Supplement: Supplementary Figures [file aging-15-205239-s001.pdf]

SUPPLEMENTARY FIGURES

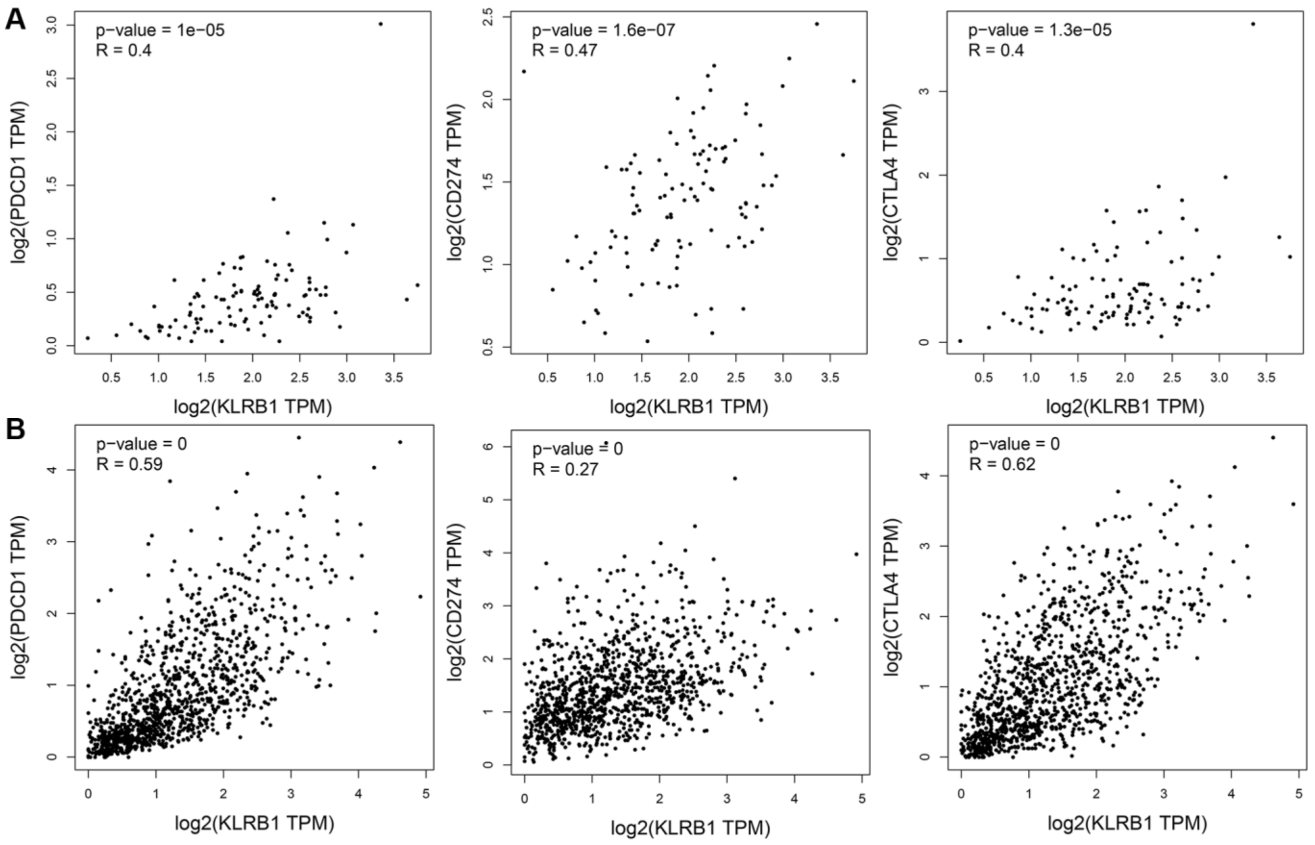

Supplementary Figure 1. KLRB1 was positively correlated with the expression of immune genes PDCD1, CD274, and CTLA4 in GEPIA database. (**A**) Normal tissues; (**B**) BRCA tissues.

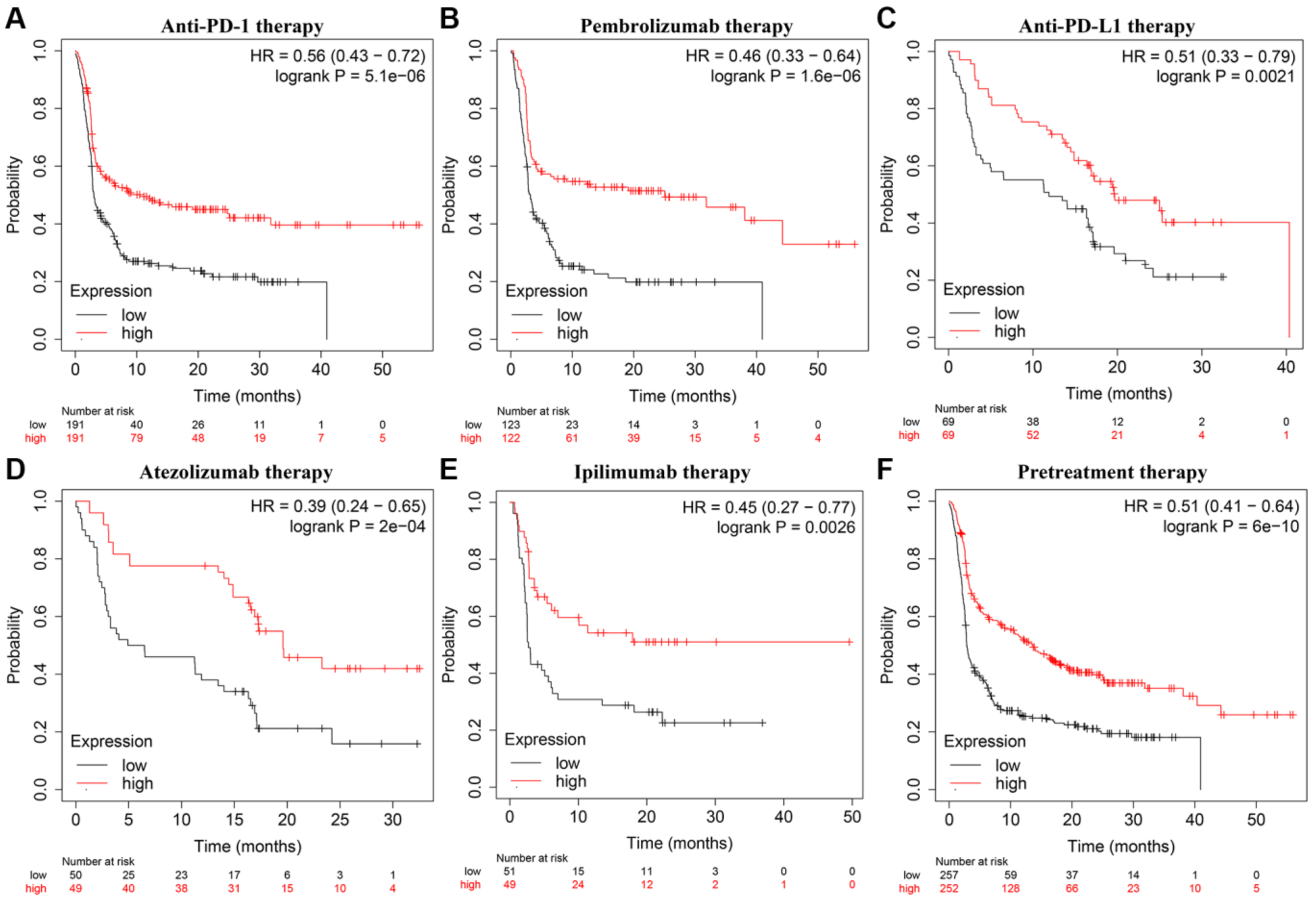

Supplementary Figure 2. KLRB1 overexpression was correlated with the PFS in cancer patients on immunotherapy. (**A**) Anti-PD-1; (**B**) Pembrolizumab; (**C**) Anti-PD-L1; (**D**) Atezolizumab; (**E**) Ipilimumab; (**F**) Pretreatment. Abbreviation: PFS: progression-free survival.
